# Context-Specific Protein Network Miner – An Online System for Exploring Context-Specific Protein Interaction Networks from the Literature

**DOI:** 10.1371/journal.pone.0034480

**Published:** 2012-04-06

**Authors:** Rajesh Chowdhary, Sin Lam Tan, Jinfeng Zhang, Shreyas Karnik, Vladimir B. Bajic, Jun S. Liu

**Affiliations:** 1 Marshfield Clinic-Marshfield Center, Marshfield Clinic Research Foundation –Biomedical Informatics Research Center, Marshfield, Wisconsin, United States of America; 2 Department of Statistics, Florida State University, Tallahassee, Florida, United States of America; 3 Computational Bioscience Research Center, King Abdullah University of Science and Technology (KAUST), Thuwal, Kingdom of Saudi Arabia; 4 Department of Statistics, Harvard University, Cambridge, Massachusetts, United States of America; Semmelweis University, Hungary

## Abstract

**Background:**

Protein interaction networks (PINs) specific within a particular context contain crucial information regarding many cellular biological processes. For example, PINs may include information on the type and directionality of interaction (e.g. phosphorylation), location of interaction (i.e. tissues, cells), and related diseases. Currently, very few tools are capable of deriving context-specific PINs for conducting exploratory analysis.

**Results:**

We developed a literature-based online system, Context-specific Protein Network Miner (CPNM), which derives context-specific PINs in real-time from the PubMed database based on a set of user-input keywords and enhanced PubMed query system. CPNM reports enriched information on protein interactions (with type and directionality), their network topology with summary statistics (e.g. most densely connected proteins in the network; most densely connected protein-pairs; and proteins connected by most inbound/outbound links) that can be explored via a user-friendly interface. Some of the novel features of the CPNM system include PIN generation, ontology-based PubMed query enhancement, real-time, user-queried, up-to-date PubMed document processing, and prediction of PIN directionality.

**Conclusions:**

CPNM provides a tool for biologists to explore PINs. It is freely accessible at http://www.biotextminer.com/CPNM/.

## Introduction

Information about protein-interaction (PI) networks (PINs) is crucial for understanding many cellular biological processes [1]. Such networks are particularly useful in elucidating cellular mechanisms that may be activated in response to, for example, environmental stimuli in normal or diseased conditions. Much of the pertinent PI information is buried in the scientific literature and cannot be retrieved in a simple and convenient manner. Moreover, much of the information relevant for PINs, e.g. type and directionality of interactions, usually is not retrieved. Recently, significant amounts of work have gone into building databases that store manually curated information on PIs from the literature. Examples of these resources include HPRD [2], MINT [3], BioGRID [4], MIPS [5], PDZBase [6], IntAct [7], STITCH [8], and others. Although the information contained in these databases is useful, the overall coverage is low, the information is not up-to-date and generally lags behind the rapidly growing literature. A complimentary approach relies on automated text-mining methods for PI extraction. These have achieved significant progress in recent years (see [9]–[12] detailing BioCreative I, II, III). These automated text-mining methods include protein name recognition [13], [14], normalized protein name extraction [15]–[25]), protein name mention normalization [26], PI-pair/triplet detection [27]–[38], and PI-sentence/abstract/method detection [39]–[46]. Together, these methods make up the foundation for integrated text-mining systems for biological applications. Some of the very few initiatives towards developing integrated text-mining based PIN extraction applications include STRING [47] and iHOP [48]. While STRING integrates information from various PI databases with PI information mined from a local, static, periodically updated copy of the PubMed database, iHOP uses a local, daily updated PubMed database.

Here we report the development of a web application we name ‘Context-specific Protein Network Miner (CPNM)’, which generates PINs in real time from the current version of the PubMed database based on a specific set of keywords provided by the user. The keywords in conjunction with the operators (AND/OR/NOT) define the specific biological context of user interest. For example, if the user wishes to generate a PIN that is specific to asthma but not diabetes, the query could be formulated as ‘asthma NOT diabetes.’ To our knowledge, there exists no other PIN generating system currently available with similar context-search capability. Compared to the existing systems, CPNM provides a combination of several unique features, making it a useful tool for biomedical research: (1) CPNM provides PI information specific to the biological context that may include interaction types and direction, related gene ontology (GO)-terms, related diseases and tissues, and other related concepts provided as input by the user; (2) CPNM's ontology-based expansion of query terms provides better coverage of the search results and an enhancement of the PubMed query capabilities; (3) online-processing of PubMed abstracts ensures consistently up-to-date search results; and (4) CPNM outputs PINs containing type and directionality of protein interactions, along with summary statistics of the interaction network, making identified PINs more useful. With CPNM, our goal is to provide a platform for researchers to gain insights into the mechanisms responsible for the functioning of cellular systems based on the identified PINs.

## Methods

### Design and Implementation

**Figure 1:**
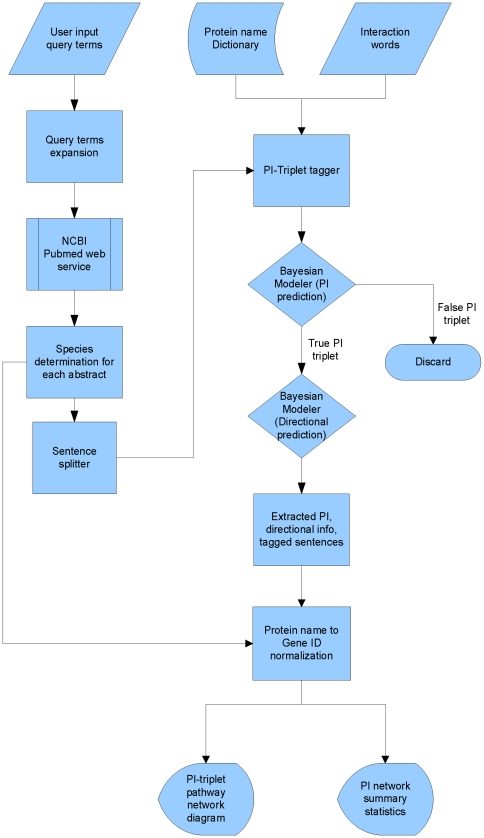
Architecture of CPNM system.

The architecture of the CPNM system is shown in Figure 1. CPNM consists of the following modules:

### 1. Search query formulation and retrieval of abstracts from the PubMed system [49]

#### 1.1 Query building

**Figure 2:**
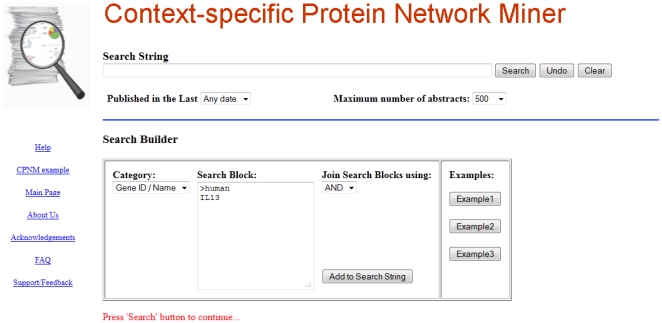
CPNM web interface with query builder.

CPNM provides a web interface that allows the user to build search queries. Search queries may contain keywords or concepts belonging to one of the following categories that are frequently used in research: diseases, proteins, GO-terms, and tissues. In addition, the user can input keywords that do not belong to any of these four categories by entering them as ‘free-text’ in the interface. The user also has the option to input species names. The query builder allows the user to separate the individual keywords in these different categories by using AND/OR/NOT operators. The interface is shown in Figure 2.

#### 1.2 Query keyword expansion

**Figure 3:**
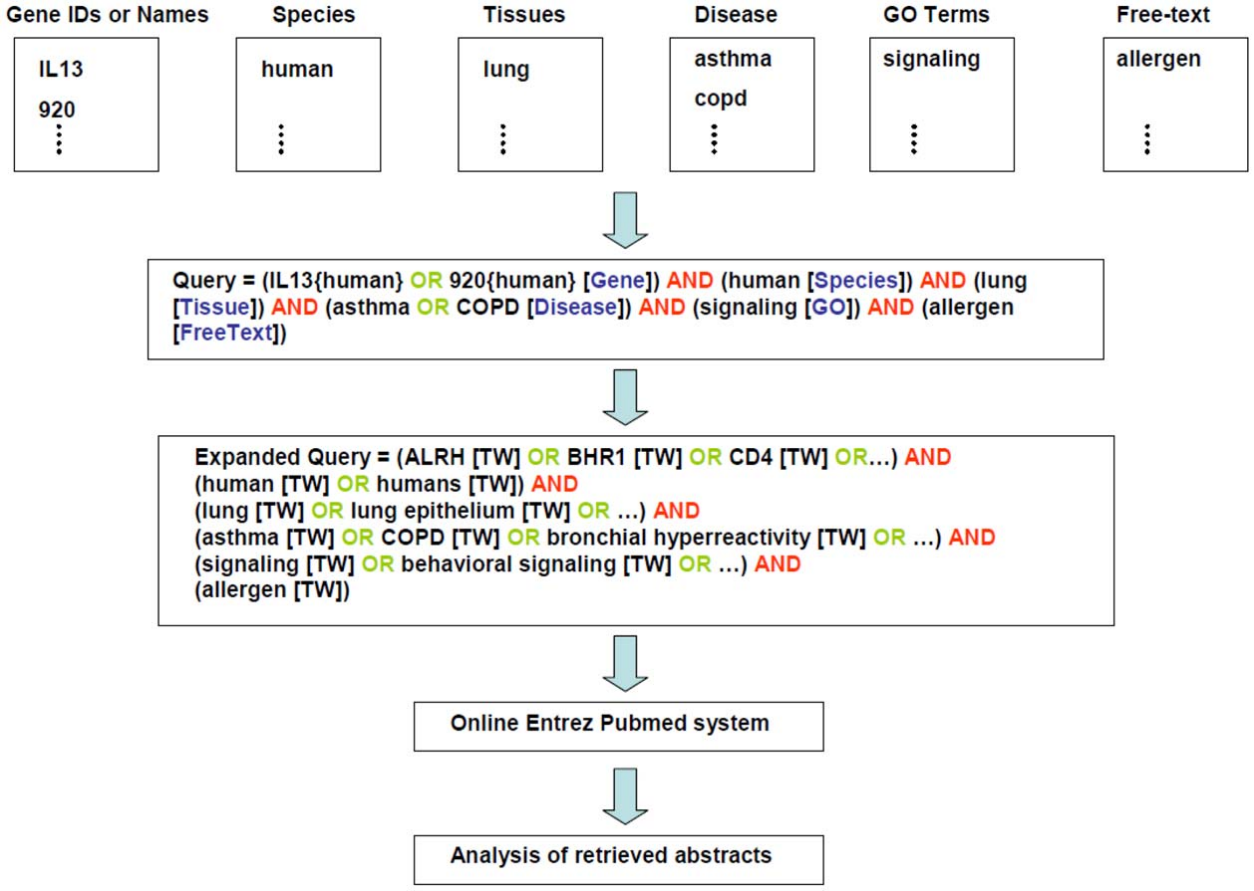
Sample query formulation by CPNM.

CPNM expands query keywords submitted by the user that belong to the categories of gene/protein names, diseases, GO-terms and tissues. This is done by retrieving all synonyms and other related terms that lie below the query keyword node (i.e. from the node up to all leaves at the first level down) in the ontology tree network provided by the Open Biological and Biomedical Ontologies (OBO) foundry [50]. The ontology trees for our target categories can be found in the following OBO foundry files: HumanDO.obo [51] for diseases, pro.obo [52] for proteins, gene_ontology_ext.obo [53] for GO-terms, and BrendaTissueOBO [54] for tissues. For proteins, we also use synonyms given in the Entrez Gene database [55]. Query keywords input to the system as ‘free-text’ are not expanded. A sample query expansion by CPNM is shown in Figure 3. Query expansion is a novel aspect of CPNM that enhances the search function of the PubMed system in our case for the purpose of PIN generation.

#### 1.3 Querying PubMed and abstract retrieval

The expanded user query is passed by CPNM to the Entrez PubMed system in real-time using their webservice to retrieve a set of abstracts that satisfy the query. CPNM searches the PubMed database using the Text Fields word tag [tw], which allows searching of most sections of an abstract, including the title, abstract body, MeSH terms, subheadings, and others. The retrieved abstracts are then processed by CPNM for PIN generation.

### 2. Sentence splitter and pre-processing

The abstracts that are retrieved from PubMed are processed locally and split into individual sentences. Further processing is done on sentences by placing the ‘space’ character before/after delimiters, such as, comma, colon, and semicolon. This is performed in CPNM by PIMiner [56].

### 3. Tagger module

The name tagger in PIMiner [56] is used for tagging occurrences of protein names and other terms (e.g. interaction words) in sentences.

#### 3.1 Protein name tagging

The PIMiner tagger uses an exhaustive dictionary containing over eight million of proteins names and their variants. The protein name dictionary was compiled by extracting data from various sources including BioThesaurus [57], UniProtKB/Swiss-Prot database [58] and NCBI Entrez Gene database. The dictionary was cleaned by filtering out commonly occurring English words and one letter/digit acronyms/short-forms.

The PIMiner tagger attempts to ‘soft’ match the maximum length substring in a sentence with the protein names in the dictionary and is optimized for processing large volumes of text in reasonable time. We convert all non-word characters in a sentence to spaces e.g. ‘$’, ‘−’, ‘+’. This enables us to match, for example, protein ‘CD2+’ in a sentence using protein name ‘CD2’. We also use general terms in soft matching of protein name, e.g. ‘beta’, ‘alpha’. If there is no ‘TGF2 beta’ but only ‘TGF2’ in our dictionary, we are still able to match ‘TGF2 beta’ as protein name in the sentence. The tagger also attempts to detect some variations in protein names by recognizing certain types of domain-specific bag-of-words ahead of the detected protein name in the sentence. For example, the tagger will be able to detect protein ‘X receptor’ in a sentence if protein ‘X receptor’ does not exist in the dictionary, but protein ‘X’ does. The tagger handles case-sensitive variations of protein names by matching single word protein names in a sentence in a case-sensitive manner and multiple-word protein names in case-insensitive manner. This is done to avoid the matching of commonly occurring single non-protein words that are most frequently written in lower case. Case-insensitivity is retained for matching protein names composed of multiple words, because there is a smaller chance of erroneously matching non-protein multiple word concepts in the text.

#### 3.2 Interaction word tagging

Our tagger uses an interaction word list that contains over 2000 unique terms, including variant forms that contain hyphens and those that represent American/British English language variations. These interaction terms describe the potential nature or type of the interaction between two interacting proteins identified in the text. Interaction words are tagged in sentences by case-insensitive string matching.

#### 3.3 User context-term tagging

All expanded user input context-terms are tagged in the text by case-insensitive string matching.

### 4. PI relationship detection/extraction module

The PI extraction module retains each tagged query sentence that contains at least one triplet, which consists of two normalized protein names and one interaction word. Feature vectors are then extracted for each triplet in the sentence and submitted to a Bayesian Network (BN) model that is trained on a dataset of manually curated triplets (for details refer to [27], [56]). The model then estimates the probability of each triplet being a true interaction.

### 5. PI direction prediction module

For predicting the directionality of interaction, the interaction words in our list are first separated into two groups, i.e. ‘with-direction’ and ‘without-direction.’ For example, words such as ‘methylates’ imply direction, while words such as ‘interacts’ imply no direction. Triplets predicted to be true interactions by the PI extraction module are assessed for any implicit direction based on the interaction words they contain. The triplets that show implicit direction are then processed to determine their actual direction. We define the direction between the two proteins in a triplet as follows: i) left→right direction means that the interaction word relationship ‘flows’ from the protein in the triplet that appears first in the sentence to the protein that appears second in the sentence while ii) left←right direction means that the interaction word relationship ‘flows’ from the protein in the triplet that appears second in the sentence to the protein that appears first in the sentence. Using the features employed in the PI extraction module (refer to [27], [56]), feature vectors are extracted for each query triplet (two proteins+interaction word) in the sentence and parsed to the BN model that is trained on a dataset of manually curated triplets/interactions with direction information. The model then estimates the probability of either of the two directions of protein interaction relationship being true. We provide online the list of interaction words ‘with-direction’ and the training data used for this step. The dataset we used for directionality prediction was randomly selected and curated by three domain experts; we went with the majority decision on the direction annotation. Interaction directionality prediction is one of the novel aspects of the present study.

### 6. Protein name mention normalization to official symbols

The protein names tagged by CPNM are normalized to their official symbols given in the Entrez Gene database. We analyze the title, body and MeSH term sections of an abstract to see if any scientific/common names of species from NCBI Taxonomy are mentioned in the text. If any species is mentioned in these sections, we associate and save the taxonomy ID of the detected species (using names.dmp file of NCBI Taxonomy FTP site) with the corresponding PubMed ID. We map (using gene_info file) protein name mentions that we recognize in the PubMed abstract to their corresponding Entrez Gene IDs using taxonomy ID that we associated with the abstract. We do case-sensitive exact match of a protein name mention against the following fields in the NCBI's gene_info file in the order: official symbol, synonym, full name, other symbol and locus tag. If a name is found in a field, the associated official gene symbol and Entrez Gene ID are retrieved, and the normalization task is completed. If, however, we cannot find protein name in any of the five fields with exact string match, we do a case-insensitive exact match and repeat the above steps. Finally, if we still cannot match a protein name, we perform the following transformation steps on protein name mentions (common variants):

- ‘XXX 1’ to ‘XXX1’
- ‘XXX receptor’ to ‘XXXR’
- ‘XXX gene’ to ‘XXX’ - remove common domain specific general phrase endings, such as, protein, gene, chain, delta, alpha, beta, gamma, epsilon from recognized protein names.

We then repeat the case-sensitive and case-insensitive string matching steps as above. Ambiguous names that we are unable to resolve using our pipeline are displayed with their gene IDs in the output (e.g. one name mapped to two gene IDs). If the protein name cannot be found, we retain the recognized protein name and show its Entrez Gene ID as blank in output. The normalization module of CPNM is a new feature that is not present in PIMiner.

### 7. PIN reporting module

PIs extracted from the text are presented to the user by CPNM in both tabular and graphical format with several different functions provided for easy navigation, viewing and exploration. PIs are reported at two levels of granularity: i) PIs that co-occur with the input keywords at the document level, and ii) PIs that co-occur with the input keywords at the sentence level. The former option is provided to increase the coverage of the results, while the latter option is provided to increase the specificity of the results. CPNM also has an option for the user to view interactions directly related to a given input protein.

#### 7.1 Individual PI reporting module

The system reports individual PIs and these are tabulated in the CPNM output along with a likelihood score, interaction type, and direction of interaction, if available. The table has sortable columns. In the table, CPNM also reports the evidence sentence and highlights the extracted PI triplet terms therein. The user-input context-terms are also shown highlighted in the reported sentence and additionally in the corresponding abstract. The output allows the user to look at the association evidence between PIs and the corresponding context-terms in the abstract. We also provide select/check boxes in the output table to allow manual selection of PIs for diagram if necessary.

In order to provide further information for the user, CPNM links protein names with associated Entrez Gene IDs reported in the output table and network graph to their corresponding pages in the Entrez Gene database. The Entrez database provides gene-centric information that may provide the user with more specific details about the target protein.

#### 7.2 PIN diagram module

CPNM reports PINs (generated from extracted PIs) in an interactive graphical form. For drawing protein network diagrams, CPNM uses a Cytoscape Web plugin [59]. By following an edge direction the user can view all proteins that are connected in the network and how they are connected. Additionally, the user can view the topology of the network and proteins forming hubs or spokes in the network. The user is also provided with the option to save or export the network diagram as an image or PDF file.

#### 7.3 PIN summary module

The protein network summary module of CPNM calculates summary statistics from the reported protein interaction network. It summarizes the PIN diagram in three different tables as follows: i) a ranked list of proteins based on the number of other proteins to which they are directly connected in the network (neighbors), indicating the hub-property of the protein; ii) a ranked list of the most directly connected protein pairs, which could be the pairs that are most well studied in literature, for example; and iii) a ranked list of proteins with the number of outbound and inbound directed edges and the number of undirected edges for each node in the network, as edge direction may give some indication about a protein's regulatory function.

#### 7.4 Filter function module

CPNM provides fine control to users by allowing them to generate PINs while applying filter functions on the date to control how recent the retrieved abstracts are, the number of relevant abstracts returned by the PubMed system, and type of interactions (e.g. methylation, phosphorylation) of interest. In addition, the user can limit the number of interactions in the PIN graph by selecting a stricter probability threshold (e.g. selecting top predictions with probability values higher than 0.95).

### Availability and requirements

Project name: CPNM web tool

Project home page: http://www.biotextminer.com/CPNM/

Operating system(s): Platform independent

Programming languages used to develop CPNM: Perl, Java, JavaScript, Cytoscape web library, NCBI E-utilities

Other requirements: Apache Webserver

Browser requirements: IE 8, Firefox 4, Safari 5, Chrome 10, Opera 11, or higher versions of these

License: Webserver is free for use for non-profit purposes

Any restrictions to use by non-academics: Contact corresponding author

Online Help pages: Provided at http://biotextminer.com/CPNM/files/CPNM-Help.pdf

## Results and Discussion

Here we describe the development of an application to mine and explore PINs related to a particular biological context. The context is defined by the user query, which is a combination of keywords and the operators that separate them. For each user query, CPNM generates a PIN based on the literature. The idea of combining a user-specific context search involving multiple biological concepts with PIN generation makes biological sense since any cellular biological-context may represent a different PIN. To our knowledge, no application with the set of features as provided by CPNM is currently available for researchers that can generate PINs from the literature. Most available PIN-generating systems allow a *single* named entity (most often a gene or protein name) to be input by the user, which is restrictive.

CPNM possesses several features that together make the system unique compared to similar web services. These include:

- Context-specificity of PINs: Each PIN generated by CPNM corresponds to a biological-context of interest that is defined by a specific set of keywords provided by the user. For example, one may be interested in extracting PINs from PubMed abstracts associated with the following set of keywords: {asthma (disease), 4790 (GeneID for NF-kappaB), human (species), epithelium (tissue), allergen (condition/event as free-text), and signalling (GO-term)}. The retrieved documents based on this set of keywords will be context-specific. Consequently, protein interactions and their network that CPNM attempts to extract from the retrieved documents are also likely to be related to the user-context. The output of CPNM includes the evidence sentence along with the associated abstract with tagged keywords for user validation.
- Flexible ontology-based query system: CPNM expands query terms using ontology that ensures higher coverage of retrieved abstracts thereby enhancing the PubMed search function.
- Real-time processing of up-to-date information: CPNM queries and processes PubMed data ‘on-the-fly’ so that results are always based on the most up-to-date version of PubMed.
- Directionality of interaction: CPNM predicts directionality of protein interactions based on interaction words, which may give some more insight into the cellular mechanisms.
- PIN reporting system and information filtering system: As detailed above, we have provided various functions in CPNM for easy exploration of PINs by the user. The user has the option to filter PIs that co-occur with the input terms at the document level for more coverage or those that co-occur only at the sentence level to be more specific. Additionally, if the user inputs a protein name, CPNM optionally allows the user to view only direct interactions involving the input protein.

The CPNM application pipeline uses various software modules related to different sub-tasks of PIN extraction and presentation. For example, CPNM uses the functionality of our previously designed system, PIMiner [56] internally for protein name tagging and protein interaction relationship prediction. Though CPNM may share some common features with PIMiner, there are marked differences in their purposes, functionality and objectives. PIMiner uses raw text as input and predicts PI-triplets and may be suitable for biocuration type of work, while CPNM uses context-indicating keywords as input and predicts protein interaction networks and may be suitable to researchers in biology and biomedical field who wish to quickly study/explore protein networks specific to a biological condition. Overall, CPNM can be thought of as a real-time plugin/extended-app to the PubMed system; though we also modify/enhance the basic search functionality provided by PubMed system. CPNM uses various previously published modules in its architecture in addition to some new modules that might be novel in their own sense (e.g. for directionality prediction, protein name normalization and protein network generation with provision of various filter/summary functions), its overall end-to-end functionality is also novel.

Tables 1, 2 and 3 summarize the performance of some of the CPNM modules. The performance of different modules appears satisfactory. It is worthwhile to note that the performance figures for the three individual modules shown in the table are based on different datasets. The performance of protein name recognition module was evaluated based on an AIMed dataset [60] while the performance of the PI-triplet recognition module was based on a manually curated dataset used in a previous study [56]. To test the performance of the module for predicting the directionality of PIs, we used data-samples from our earlier study [56] that contained true PI-triplets with direction and added to the set a few more manually curated samples chosen randomly from the literature.

**Table 1:** Accuracy of CPNM on gene/protein name tagging task using holdout test datasets from AIMed and BioCreative.

	Recall (%)	Precision (%)	F-measure (%)
On AIMed data (recognition)	79	68.8	73.6
On BioCreative II GN task dataset (normalization)	81	54.5	65.2

**Table 2:** Accuracy of CPNM on PI triplet prediction task based on 10-fold cross validation on a gold-standard dataset.

With training data class distribution as: 668 true triplet samples and 1882 false triplet samples				
Class	Precision (%)	Recall (%)	F-Measure (%)	ROC Area (%)
for true triplet class	72.7	75.4	74.1	91

**Table 3:** Accuracy of CPNM on PI directionality prediction task based on 10-fold cross validation on a gold-standard dataset.

With training data class distribution as: 116 samples with left→right direction and 29 samples with left←right direction				
Class	Precision (%)	Recall (%)	F-Measure (%)	ROC Area (%)
left→right	95.7	96.6	96.1	93.3
left←right	86.2	83.3	84.7	93.3

For protein name recognition and normalization task, we evaluated our system on AIMED and BioCreative II GN task datasets, the results of this evaluation are presented in Table 1. In our experience, AIMed appears to be more accurately annotated dataset for protein names compared to the BioCreative II dataset. We show in Table S1, a small sample of protein name mentions that CPNM detected which were not annotated as proteins in the BioCreative II dataset key. Such cases lead to lower precision for our system. It is worthwhile to note that CPNM attempts to normalize each protein name mention recognized by it in the input text. Therefore it may not be appropriate to evaluate its performance on BioCreative II GN task since this task is about reporting only the normalized forms of protein names present in an abstract with no consideration given to recognition of actual name mentions. For example, if a protein is mentioned several times in an abstract possibly in variant forms, BioCreative II GN task in its evaluation only focusses on detection of any one of these variants in normalized form, not all. In Tables S2, S3, S4 and Figure S1 respectively, we show that CPNM functionality/performance compares favourably with some of the state of the art programs (NLProt [26], GNAT [25], LAITOR [38]) in protein name recognition/normalization and protein interaction detection.

Regarding efficiency of the entire CPNM pipeline, we found in our internal tests that CPNM takes about 104 sec to process 500 abstracts and generate a PIN for a specific query; similarly CPNM takes about 43 sec to process 50 abstracts. This time includes time required to retrieve abstracts in real-time from PubMed and the time required to process the data. Therefore, time taken for processing may vary depending on the user-query and number of abstracts selected for analysis. In general, the larger the number of abstracts requested from PubMed the longer the time CPNM requires to download PubMed abstracts and process the text; where time for retrieving abstracts is generally much more than the actual processing.

**Figure 4:**
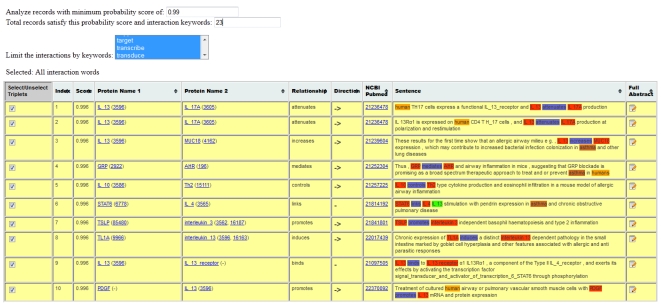
CPNM output showing protein interactions extracted from literature for Case Study I.

**Figure 5:**
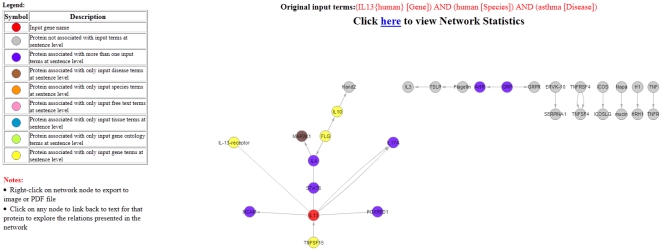
PIN generated by CPNM for Case Study I.

**Figure 6:**
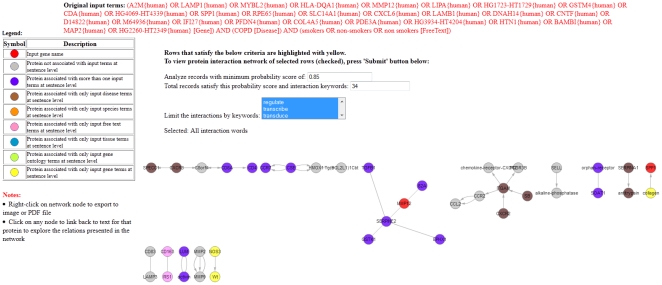
PIN generated by CPNM for Case Study II.

**Figure 7:**
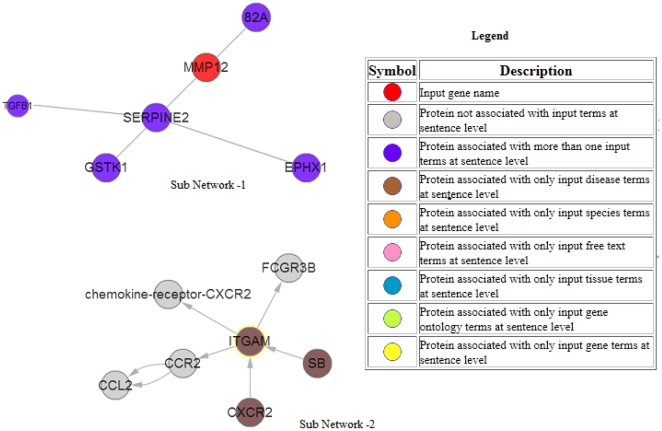
Hub nodes in PIN generated by CPNM for Case Study II.

CPNM usage examples: In this section we describe two sample case studies illustrating the use of CPNM:

- **Case Study I**: To extract a PIN from literature associated with IL13 gene in human asthma, we pass the following query to CPNM: (IL13{human} [gene]) AND (human [Species]) AND (asthma [Disease]). We restrict the number of abstracts to 500. The extracted PIs are shown in Figure 4 (with probability threshold of 0.99 being used). The PIN generated by CPNM for this query is shown in Figure 5 and the related statistics are presented in Tables 4, 5 and 6. Using the generated PIN, we collected and analyzed all hub node proteins in the network. We define hub nodes as those that had two or more neighbors in the network. Since hub-node proteins potentially could carry important information about the target context, we investigated further their membership in terms of their pathway interaction/membership.
  The proteins that satisfied the hub-protein criteria of having more than two neighbors in the PIN included: IL13, IL4, FLG, GRP, IL10, STAT6, and TSLP. We then selected these hub node proteins and queried them against the pathway database, hiPathDB [61]. This database integrates several well-known pathway databases, such as, KEGG [62], NCI-nature [63], BioCarta (http://www.biocarta.com) and Reactome [64]. The pathway involvement of these hub node proteins that we obtained from hiPathDB database is presented in Table 7.
  From the retrieved pathway information involving our hub-proteins, we found through manual verification of individual pathway sources in hiPathDB that there were some pathways in our list that were previously associated with our context disease term, asthma. These include Jak-STAT signaling pathway, Cytokine-cytokine receptor signaling pathway, Calcineurin-regulated NFAT pathway, GATA3 related th2 cytokine pathway (refer Table 7). Thus, using CPNM we were able to connect the context with the pathway information via information derived from the generated PIN. We also found several other pathways, however, their association with asthma could not be verified. Such novel candidate associations between query context and pathways may be interesting candidate hypotheses worth exploring further using other methods.
  In our analysis all our hub-proteins, except FLG (Filaggrin), show up as a part of some pathway (refer Table 7). FLG is a protein that shows up in our target PIN as associated with input gene IL13. We searched PubMed to see if FLG has been implicated in asthma and we found that FLG gene has been associated with the risk of asthma [65]–[68] although we also found some evidence that pointed otherwise [69]. Another hub-protein, GRP, in our network appears to be undergoing investigation [70] as an anti-inflammatory therapeutic agent for asthma (currently investigated in mice). Since CPNM operates real time, it is able to capture such current information from PubMed.
  Overall, CPNM can be explored by users as a complimentary tool for validating known hypothesis or to generate novel ones related to a biological context (e.g. gene, disease) to have further insights into associated molecular mechanisms.
- **Case Study II**: In this case study we use CPNM to a generate context specific PIN associated with differentially expressed genes (up/down regulated genes) in a gene expression experiment.
  Gene expression experiments generate a lot of valuable data in a high throughput manner. One typical challenging problem interesting to researchers is how to elucidate and explore PINs and their topologies associated with gene expression data. In this example we show how CPNM could be used for the purpose.
  We select a gene expression experiment data (GSE3212) from our in-house collection of GEO datasets for common respiratory diseases; the database can be accessed at http://www.respiratorygenomics.com/GeneExpression/. This series (GSE3212) compares gene expression in alveolar macrophages of smokers and non-smokers in patients with chronic obstructive pulmonary disease (COPD) [71]. In this case study we selected genes in this dataset that were either up (11 genes) or down (17) regulated with a fold change of three or more. Table 8 lists genes qualifying this criterion.
  We then formulated a query by using context specific information from this series such as COPD (disease name), smokers and non-smokers along with 28 differentially expressed genes. The formulated query was: {(gene names separated by OR) AND (COPD[Disease]) AND (smokers OR non-smokers OR nonsmokers[FreeText]). We passed the query to CPNM that extracted a PIN (using a threshold of 0.85). Snapshot of the query and the results returned are shown in Figures 6 and 7.
  In the generated PIN, we found two hub node proteins that might be worth investigating further in the context of the experiment. These were ITGAM and SERPINE2, which were not part of the gene set input to CPNM. This example shows how using CPNM we were able to elucidate PIN/hub-proteins associated with a target gene expression experiment. The PINs generated this way are literature based and thus may include genes that are not part of the input differentially expressed gene set. Thus CPNM may provide a broader/bigger picture that might be associated with the target gene expression experiment. Such information can prove valuable to researchers performing gene expression experiments for investigating underlying biological mechanisms associated with diseases/drugs for example.

**Table 4:** Node neighbour (hub-protein) statistics in the network diagram (Figure 5).

Protein	Neighbours	Percent coverage (#neighbours/#total network nodes)
IL13	6	20.00%
IL4	3	10.00%
FLG	2	6.67%
GRP	2	6.67%
IL10	2	6.67%
STAT6	2	6.67%
TSLP	2	6.67%

This table shows that IL13, IL4, FLG, GRP, IL10, STAT6, and TSLP may be important hub-proteins in the network for the target biological context (IL13, asthma, human). Only nodes with two or more neighbours are shown.

**Table 5:** Evidence (edge) strength between network protein pairs shown in Figure 5 (more links/edges between two nodes typically would mean more support in the literature).

Protein Name 1	Protein Name 2	# links
IL17A	IL13	2
IL4	MAPK21	1
IL4	STAT6	1
IL4	FLG	1
AHR	GRP	1
FOXRED1	IL13	1
GRPR	GRP	1
IL13	TSLP	1
IL13	STAT6	1

Only links associated with hub-node proteins (refer Table 4) are shown.

**Table 6:** Outbound, inbound, and undirected edge connectivity for a node.

Protein	Outward	Inward	Undirected
IL13	4	1	2
IL4	1	1	1
FLG	2	0	0
GRP	2	0	0
IL10	1	1	0
TSLP	1	1	0
STAT6	0	0	2

This table shows the distribution of direction information for a given protein in the network diagram shown in Figure 5. Only links associated with hub-node proteins (refer Table 4) are shown.

**Table 7:** Pathway involvement of the hub-node proteins in the context specific network generated by CPNM in Case Study I using pathway information given in hiPathDB database [61].

Pathway ID	Pathway Name	Total Interactions	Source	Participating proteins from CPNM	Asthma association based on references provided in pathway annotation in the sources.
pid_p_200014_il4_2pathway	IL4-mediated signaling events	62	Nci-Nature	IL4,IL10,STAT6	No documented association.
path:hsa04630	**Jak-STAT signaling pathway**	9	KEGG	STAT6	Part of KEGG asthma pathway.
pid_p_200036_nfat_tfpathway	**Calcineurin-regulated NFAT-dependent transcription in lymphocytes**	8	Nci-Nature	IL4	PMID: 12452838
pid_p_100157_gata3pathway	**gata3 participate in activating the th2 cytokine genes expression**	7	BioCarta	IL4,IL13	Association with asthma documented in pathway annotation.
path:hsa04060	**Cytokine-cytokine receptor interaction**	5	KEGG	IL4,IL13,IL10,TSLP	Part of KEGG asthma pathway.
pid_p_200070_reg gr_pathway	Glucocorticoid receptor regulatory network	5	Nci-Nature	IL4,IL13	No documented association.
path:hsa05140	Leishmaniasis	4	KEGG	IL4,IL10	No documented association.
pid_p_200031_l12_2pathway	IL12-mediated signaling events	3	Nci-Nature	STAT6,IL4	No documented association.
path:hsa05142	Chagas disease	2	KEGG	IL10	No documented association.
pid_p_100134_il10pathway	il-10 anti-inflammatory signaling pathway	2	BioCarta	IL10	No documented association.
pid_p_200027_cd40_pathway	CD40/CD40L signaling	2	Nci-Nature	IL4	No documented association.
pid_p_200182_il_2_stat4pathway	IL12 signaling mediated by STAT4	2	Nci-Nature	IL4,STAT6	No documented association.
pid_p_200002_smad2_3nuclearpathway	Regulation of nuclear SMAD2/3 signaling	1	Nci-Nature	IL10	No documented association.
pid_p_200148_il2_stat5pathway	IL2 signaling events mediated by STAT5	1	Nci-Nature	IL4	No documented association.
pid_p_200149_tcrcalciumpathway	Calcium signaling in the CD4+ TCR pathway	1	Nci-Nature	IL4	No documented association.
Downstream_signal_transduction	Downstream signal transduction	1	Reactome	STAT6	No documented association.
Peptide_ligand_binding_receptors	Peptide ligand-binding receptors	1	Reactome	GRP	No documented association.

Highlighted in bold are the pathways that are known to be associated with asthma as per annotation provided in the source databases in hiPathDB.

**Table 8:** List of differentially expressed genes with fold change >3 selected for Case Study II.

Gene IDs	Regulation
A2M,LAMP1,MYBL2,HLA-DQA1,MMP12,LIPA,HG1723-HT1729,GSTM4,CDA,HG4069-HT4339,SPP1	Up regulated with fold change >3
RPE65,SLC14A1,CXCL6,LAMB1,DNAH14,CNTF,D14822,M64936,IFI27,PFDN4,COL4A5,PDE3A,HG3934-HT4204,HTN1,BAMBI,MAP2,HG2260-HT2349	Down regulated with fold change >3

In summary, we developed a versatile PubMed plugin application for real-time extraction of context-specific PINs from PubMed abstracts. We hope that CPNM will serve as a useful complimentary resource to existing PI resources. In future, to improve CPNM's functionality further, we plan to explore the following: i) integrate other third party tools (e.g. gene taggers and pathway databases) with CPNM pipeline; ii) develop automatic method for easy summarization and interpretation of the PI type and directionality information at the network level; and iii) work with a local daily-updated copy of PubMed database with good search functions and unlimited number of PubMed abstracts retrieval.

## Supporting Information

Figure S1 **Sample output of LIATOR program.**
(TIF)

Table S1 **Error analysis of CPNM on BioCreative II GN task.**
(DOC)

Table S2 **Comparison of PIMiner and NLProt.**
(DOC)

Table S3 **Comparison of CPNM and GNAT.**
(DOC)

Table S4 **Comparison of CPNM and LAITOR.**
(DOC)

## References

[1] Rzhetsky A, Seringhaus M, Gerstein M (2008). Seeking a new biology through text mining.. Cell.

[2] Keshava P, Goel R, Kandasamy K, Keerthikumar S, Kumar S (2009). Human Protein Reference Database–2009 update.. Nucleic Acids Res.

[3] Ceol A, Chatr AA, Licata L, Peluso D, Briganti L (2010). MINT, the molecular interaction database: 2009 update.. Nucleic Acids Res.

[4] Stark C, Breitkreutz BJ, Reguly T, Boucher L, Breitkreutz A (2006). BioGRID: a general repository for interaction datasets.. Nucleic Acids Res.

[5] Pagel P, Kovac S, Oesterheld M, Brauner B, Dunger-KI (2005). The MIPS mammalian protein-protein interaction database.. Bioinformatics.

[6] Beuming T, Skrabanek L, Niv MY, Mukherjee P, Weinstein H (2005). PDZBase: a protein-protein interaction database for PDZ-domains.. Bioinformatics.

[7] Aranda B, Achuthan P, Alam-FY, Armean I, Bridge A (2010). The IntAct molecular interaction database in 2010.. Nucleic Acids Res.

[8] Kuhn M, Mering C, Campillos M, Jensen LJ, Bork P (2008). STITCH: interaction networks of chemicals and proteins.. Nucleic Acids Res.

[9] Hirschman L, Colosimo M, Morgan A, Yeh A (2005). Overview of BioCreAtIvE task 1B: normalized gene lists.. BMC Bioinformatics.

[10] Smith L, Tanabe LK, Ando RJ, Kuo CJ, Chung IF (2008). Overview of BioCreative II gene mention recognition.. Genome Biology.

[11] Morgan AA, Lu Z, Wang X, Cohen AM, Fluck J (2008). Overview of BioCreative II gene normalization.. Genome Biology.

[12] Krallinger M, Vazquez M, Leitner F, Salgado D, Aryamontri AC (2011). The Protein-Protein Interaction tasks of BioCreative III: classification/ranking of articles and linking bio-ontology concepts to full text.. BMC Bioinformatics.

[13] Tsuruoka Y, Tateishi Y, Kim JD, Ohta T, McNaught J (2005). Developing a Robust Part-of-Speech Tagger for Biomedical Text, Advances in Informatics.. http://www-tsujii.is.s.u-tokyo.ac.jp/~tsuruoka/papers/pci05.pdf.

[14] Leaman R, Gonzalez G (2008). BANNER: an executable survey of advances in biomedical named entity recognition.. Pac Symp Biocomput.

[15] Okazaki N, Cho HC, Sætre R, Pyysalo S, Ohta T (2010). The gene normalization and intractive systems of the University of Tokyo in the BioCreative III challenge.. In the Proceedings of BioCreative III.

[16] Hakenberg J, Plake C, Leaman R, Schroeder M, Gonzalez G (2008). Inter-species normalization of gene mentions with GNAT.. Bioinformatics.

[17] Rebholz-SD, Arregui M, Gaudan S, Kirsch H, Jimeno A (2008). Text processing through Web services: calling Whatizit.. Bioinformatics.

[18] Hanisch D, Fundel K, Mevissen HT, Zimmer R, Fluck J (2005). ProMiner: rule-based protein and gene entity recognition.. BMC Bioinformatics.

[19] Xu H, Fan JW, Hripcsak G, Mendonça EA, Markatou M (2007). Gene symbol disambiguation using knowledge-based profiles.. Bioinformatics.

[20] Solt I, Gerner M, Thomas P, Nenadic G, Bergman CM (2010). Gene mention normalization in full texts using GNAT and LINNAEUS.. BioCreative III Workshop.

[21] Wermter J, Tomanek K, Hahn U (2009). High-performance gene name normalization with GENO.. Bioinformatics.

[22] Neves ML, Carazo JM, Pascual-MA (2010). Moara: a Java library for extracting and normalizing gene and protein mentions.. BMC Bioinformatics.

[23] Wei CH, Huang IC, Hsu YY, Kao HY (2009). http://www.computer.org/portal/web/csdl/doi/10.1109/BIBE.2009.41.

[24] Huang M, Liu J, Zhu X (2011). GeneTUKit: a software for document-level gene normalization.. Bioinformatics.

[25] Hakenberg J, Gerner M, Haeussler M, Solt I, Plake C (2011). The GNAT library for local and remote gene mention normalization.. Bioinformatics.

[26] Mika S, Rost B (2004). NLProt: extracting protein names and sequences from papers.. Nucleic Acids Res.

[27] Chowdhary R, Zhang J, Liu JS (2009). Bayesian inference of protein-protein interactions from biological literature.. Bioinformatics.

[28] Saetre R, Sagae K, Tsujii J (2008). Syntactic features for protein-protein interaction extraction.. http://www.cs.cmu.edu/~sagae/docs/saetre-lbm2007.pdf.

[29] Hunter L, Lu Z, Firby J, Baumgartner WA, Johnson HL (2008). OpenDMAP: An open source, ontology-driven concept analysis engine, with application to capturing knowledge regarding protein transport, protein interactions and cell-type-specific gene expression.. BMC Bioinformatics.

[30] Iossifov I, Rodriguez ER, Mayzus I, Millen KJ, Rzhetsky A (2009). Looking at cerebellar malformations through text-mined interactomes of mice and humans.. PLoS Comput Biol.

[31] Wren JD, Bekeredjian R, Stewart JA, Shohet RV, Garner HR (2004). Knowledge discovery by automated identification and ranking of implicit relationships.. Bioinformatics.

[32] Saetre R, Yoshida K, Miwa M, Matsuzaki T, Kano Y (2010). Extracting protein interactions from text with the unified AkaneRE event extraction system.. IEEE/ACM Trans Comput Biol Bioinform.

[33] Björne J, Ginter F, Pyysalo S, Tsujii J, Salakoski T (2010). Complex event extraction at PubMed scale.. Bioinformatics.

[34] Wong L, Liu G (2010). Protein interactome analysis for countering pathogen drug resistance.. J Comp Sci Tech.

[35] Bui QC, Katrenko S, Sloot PM (2011). A hybrid approach to extract protein-protein interactions.. Bioinformatics.

[36] Tikk D, Thomas P, Palaga P, Hakenberg J, Leser U (2010). A comprehensive benchmark of kernel methods to extract protein-protein interactions from literature.. PLoS Comput Biol.

[37] Gerner M, Nenadic G, Bergman CM (2010). An Exploration of Mining Gene Expression Mentions and their Anatomical Locations from Biomedical Text.. http://personalpages.manchester.ac.uk/postgrad/martin.gerner/pdf/getm-paper.pdf.

[38] Barbosa-Silva A, Soldatos TG, Magalhães IL, Pavlopoulos GA, Fontaine JF (2010). LAITOR–Literature Assistant for Identification of Terms co-Occurrences and Relationships.. Bioinformatics.

[39] Kim S, Shin SY, Lee IH, Kim SJ, Sriram R (2008). PIE: an online prediction system for protein-protein interactions from text.. Nucleic Acids Res.

[40] Dogan R, Yang Y, Neveol A, Huang M, Lu Z (2010). Identifying protein-protein interactions in biomedical text articles.. BioCreative III.

[41] Agarwal S, Liu F, Li Z, Yu H (2010). Machine learning based approaches for Biocreative III tasks.. BioCreative III.

[42] Fontaine JF, Navarro MA (2010). Fast classification of scientific abstracts related to protein-protein interaction using a Naive Bayesian linear classifier.. BioCreative III.

[43] Kim S, Wilbur WJ (2010). Improving protein-protein interaction article classification performance by utilizing grammatical relations.. BioCreative III.

[44] Leaman R, Sullivan R, Gonzalez G (2010). A top-down approach for finding interaction detection methods.. BioCreative III.

[45] Lourenco A, Conover M, Wong A, Pan F, Abi-Haidar A (2010). Testing Extensive Use of NER tools in Article Classification and a Statistical Approach for Method Interaction Extraction in the Protein-Protein Interaction Literature.. BioCreative III.

[46] Matos S, Campos D, Oliveira JL (2010). Vector-space models and terminologies in gene normalization and document classification.. BioCreative III.

[47] Szklarczyk D, Franceschini A, Kuhn M, Simonovic M, Roth A (2011). The STRING database in 2011: functional interaction networks of proteins, globally integrated and scored.. Nucleic Acids Res.

[48] Hoffmann R, Valencia A (2004). A gene network for navigating the literature.. Nature Genetics.

[49] Sayers EW, Barrett T, Benson DA, Bolton E, Bryant SH (2010). Database resources of the National Center for Biotechnology Information.. Nucleic Acids Res.

[50] Smith B, Ashburner M, Rosse C, Bard J, Bug W (2007). The OBO Foundry: coordinated evolution of ontologies to support biomedical data integration.. Nat Biotechnol.

[51] Du P, Feng G, Flatow J, Song J, Holko M (2009). From disease ontology to disease-ontology lite: statistical methods to adapt a general-purpose ontology for the test of gene-ontology associations.. Bioinformatics.

[52] Natale DA, Arighi CN, Barker WC, Blake J, Chang TC (2007). Framework for a Protein Ontology.. BMC Bioinformatics.

[53] Ashburner M, Ball CA, Blake JA, Botstein D, Butler H (2000). Gene Ontology: tool for the unification of biology. The Gene Ontology Consortium.. Nature Genet.

[54] Gremse M, Chang A, Schomburg I, Grote A, Scheer M (2011). The BRENDA Tissue Ontology (BTO): the first all-integrating ontology of all organisms for enzyme sources.. Nucleic Acids Res.

[55] Maglott D, Ostell J, Pruitt KD, Tatusova T (2007). Entrez Gene: gene-centered information at NCBI.. Nucleic Acids Res.

[56] Chowdhary R, Zhang J, Tan SL, Osborne D, Bajic VB PIMiner: a web tool for extraction of Protein Interactions from Biomedical Literature.. International Journal of Data Mining and Bioinformatics (IJDMB).

[57] Liu H, Hu ZZ, Zhang J, Wu C (2006). BioThesaurus: a web-based thesaurus of protein and gene names.. Bioinformatics.

[58] Apweiler R, Bairoch A, Wu CH, Barker WC, Boeckmann B (2004). UniProt: the Universal Protein knowledgebase.. Nucleic Acids Res.

[59] Lopes CT, Franz M, Kazi F, Donaldson SL, Morris Q (2010). Cytoscape Web: an interactive web-based network browser.. Bioinformatics.

[60] Bunescu R, Ge R, Kate RJ, Marcotte EM, Mooney RJ (2005). Comparative experiments on learning information extractors for proteins and their interactions.. Artificial Intelligence in Medicine.

[61] Yu N, Seo J, Rho K, Jang Y, Park J (2012). hiPathDB: a human-integrated pathway database with facile visualization.. Nucleic Acids Research.

[62] Kanehisa M, Goto S, Sato Y, Furumichi M, Tanabe M (2012). KEGG for integration and interpretation of large-scale molecular data sets.. Nucleic Acids Research.

[63] Schaefer CF, Anthony K, Krupa S, Buchoff J, Day M (2009). PID: the Pathway Interaction Database.. Nucleic Acids Research.

[64] Croft D, O'Kelly G, Wu G, Haw R, Gillespie M (2011). Reactome: a database of reactions, pathways and biological processes.. Nucleic Acids Research.

[65] Palmer CNA, Ismail T, Lee SP, Terron-Kwiatkowski A, Zhao Y (2007). Filaggrin null mutations are associated with increased asthma severity in children and young adults.. Journal of Allergy and Clinical Immunology.

[66] Poninska J, Samolinski B, Tomaszewska A, Raciborski F, Samel-Kowalik P (2011). Filaggrin Gene Defects Are Independent Risk Factors for Atopic Asthma in a Polish Population: A Study in ECAP Cohort.. PLoS ONE.

[67] Schuttelaar MLA, Kerkhof M, Jonkman MF, Koppelman GH, Brunekreef B (2009). Filaggrin mutations in the onset of eczema, sensitization, asthma, hay fever and the interaction with cat exposure.. Allergy.

[68] Weidinger S, O'Sullivan M, Illig T, Baurecht H, Depner M (2008). Filaggrin mutations, atopic eczema, hay fever, and asthma in children.. Journal of Allergy and Clinical Immunology.

[69] Rogers AJ, Celedón JC, Lasky-Su JA, Weiss ST, Raby BA (2007). Filaggrin mutations confer susceptibility to atopic dermatitis but not to asthma.. Journal of Allergy and Clinical Immunology.

[70] Zhou S, Potts EN, Cuttitta F, Foster WM, Sunday ME (2011). Gastrin-releasing peptide blockade as a broad-spectrum anti-inflammatory therapy for asthma.. Proceedings of the National Academy of Sciences.

[71] Heguy A, O'Connor T, Luettich K, Worgall S, Cieciuch A (2006). Gene expression profiling of human alveolar macrophages of phenotypically normal smokers and nonsmokers reveals a previously unrecognized subset of genes modulated by cigarette smoking.. Journal of Molecular Medicine.

