# Supplementary figures and images for: Context-Specific Protein Network Miner – An Online System for Exploring Context-Specific Protein Interaction Networks from the Literature
Source: PLoS One. 2012 Apr 6;7(4):e34480. doi: 10.1371/journal.pone.0034480 (PMC3321019; doi:10.1371/journal.pone.0034480)

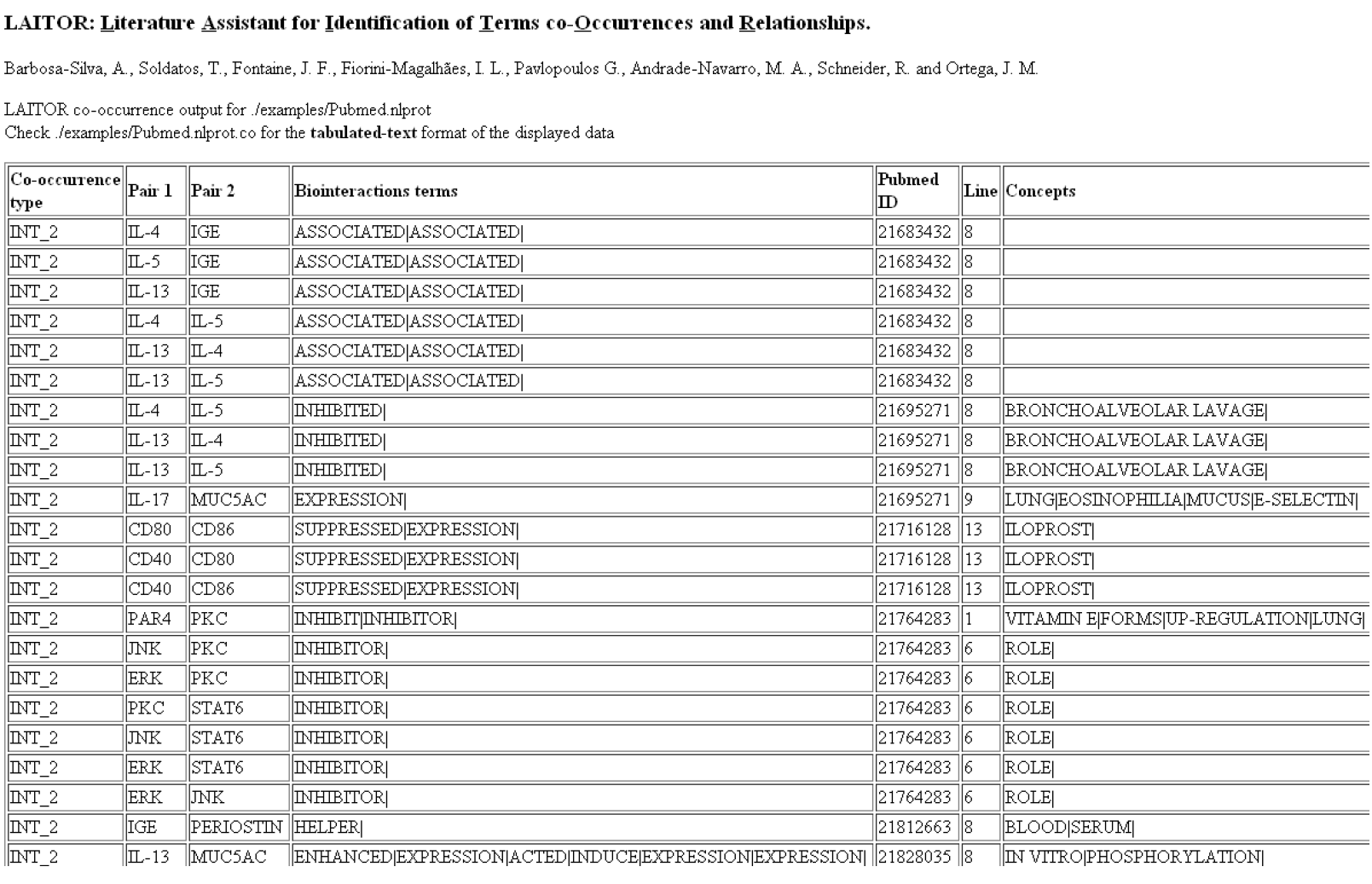

Supplement: Figure S1 — Sample output of LIATOR program. (TIF) [file pone.0034480.s001.tif]
