# Supplementary material for: Context-Specific Protein Network Miner – An Online System for Exploring Context-Specific Protein Interaction Networks from the Literature
Source: PLoS One. 2012 Apr 6;7(4):e34480. doi: 10.1371/journal.pone.0034480 (PMC3321019; doi:10.1371/journal.pone.0034480)
Supplement: Table S1 — Error analysis of CPNM on BioCreative II GN task. (DOC) [file pone.0034480.s002.doc]

**Table S1: Error analysis of CPNM on BioCreative II GN task[[1]](#endnote-2).**

| **Index** | **Pubmed ID** | **Gene ID** | **Gene Name** | **Sentence** |
| --- | --- | --- | --- | --- |
| 1 | [10072587](http://www.ncbi.nlm.nih.gov/pubmed/?term=10072587) | [708](http://www.ncbi.nlm.nih.gov/sites/entrez?cmd=search&db=gene&term=708%5Buid%5D); [972](http://www.ncbi.nlm.nih.gov/sites/entrez?cmd=search&db=gene&term=972%5Buid%5D); [4050](http://www.ncbi.nlm.nih.gov/sites/entrez?cmd=search&db=gene&term=4050%5Buid%5D) | [p33](http://www.ncbi.nlm.nih.gov/gene?term=p33) | Although the significance of this observation with respect to carcinogenesis remains to be established, the data suggest that ING1L might be involved in colon cancers through interference with signal(s) transmitted through p53 and p33(ING1). |
| 2 | [10092817](http://www.ncbi.nlm.nih.gov/pubmed/?term=10092817) | [959](http://www.ncbi.nlm.nih.gov/sites/entrez?cmd=search&db=gene&term=959%5Buid%5D) | [IgM](http://www.ncbi.nlm.nih.gov/gene?term=IgM) | Ligation of human monocytes with immobilized R3, a IgM mAb recognizing C1qRP, also triggers enhanced phagocytic capacity of these cells in the absence of ligand, verifying the direct involvement of this polypeptide in the regulation of phagocytosis. |
| 3 | [10092817](http://www.ncbi.nlm.nih.gov/pubmed/?term=10092817) | [50639](http://www.ncbi.nlm.nih.gov/sites/entrez?cmd=search&db=gene&term=50639%5Buid%5D) | [MBL](http://www.ncbi.nlm.nih.gov/gene?term=MBL) | C1q, mannose-binding lectin (MBL), and pulmonary surfactant protein A (SPA) interact with human monocytes and macrophages, resulting in the enhancement of phagocytosis of suboptimally opsonized targets. mAbs that recognize a cell surface molecule of 126,000 Mr, designated C1qRP, have been shown to inhibit C1q- and MBL-mediated enhancement of phagocytosis. |
| 4 | [10092817](http://www.ncbi.nlm.nih.gov/pubmed/?term=10092817) | [912](http://www.ncbi.nlm.nih.gov/sites/entrez?cmd=search&db=gene&term=912%5Buid%5D) | [R3](http://www.ncbi.nlm.nih.gov/gene?term=R3) | Ligation of human monocytes with immobilized R3, a IgM mAb recognizing C1qRP, also triggers enhanced phagocytic capacity of these cells in the absence of ligand, verifying the direct involvement of this polypeptide in the regulation of phagocytosis. |
| 5 | [10215850](http://www.ncbi.nlm.nih.gov/pubmed/?term=10215850) | [675](http://www.ncbi.nlm.nih.gov/sites/entrez?cmd=search&db=gene&term=675%5Buid%5D); [2177](http://www.ncbi.nlm.nih.gov/sites/entrez?cmd=search&db=gene&term=2177%5Buid%5D); [5663](http://www.ncbi.nlm.nih.gov/sites/entrez?cmd=search&db=gene&term=5663%5Buid%5D) | [FAD](http://www.ncbi.nlm.nih.gov/gene?term=FAD) | It is highly homologous to the previously described cytosolic enzyme (TrxR1), including the conserved active site CVNVGC and the FAD-binding and NADPH-binding domains. |
| 6 | [10215850](http://www.ncbi.nlm.nih.gov/pubmed/?term=10215850) | [1666](http://www.ncbi.nlm.nih.gov/sites/entrez?cmd=search&db=gene&term=1666%5Buid%5D) | [NADPH](http://www.ncbi.nlm.nih.gov/gene?term=NADPH) | It is highly homologous to the previously described cytosolic enzyme (TrxR1), including the conserved active site CVNVGC and the FAD-binding and NADPH-binding domains. |
| 7 | [10215850](http://www.ncbi.nlm.nih.gov/pubmed/?term=10215850) | [25824](http://www.ncbi.nlm.nih.gov/sites/entrez?cmd=search&db=gene&term=25824%5Buid%5D) | [thioredoxin reductase](http://www.ncbi.nlm.nih.gov/gene?term=thioredoxin reductase) | We have isolated a 1918-bp cDNA from a human adrenal cDNA library which encodes a novel thioredoxin reductase (TrxR2) of 521 amino acid residues with a calculated molecular mass of 56.2 kDa. |
| 8 | [10235266](http://www.ncbi.nlm.nih.gov/pubmed/?term=10235266) | [23237](http://www.ncbi.nlm.nih.gov/sites/entrez?cmd=search&db=gene&term=23237%5Buid%5D) | [ARC](http://www.ncbi.nlm.nih.gov/gene?term=ARC) | The DRIPs are almost indistinguishable from components of another new cofactor complex called ARC, which is recruited by other types of transcription activators to mediate transactivation on chromatin-assembled templates. |
| 9 | [10235266](http://www.ncbi.nlm.nih.gov/pubmed/?term=10235266) | [1264](http://www.ncbi.nlm.nih.gov/sites/entrez?cmd=search&db=gene&term=1264%5Buid%5D); [6876](http://www.ncbi.nlm.nih.gov/sites/entrez?cmd=search&db=gene&term=6876%5Buid%5D) | [SMCC](http://www.ncbi.nlm.nih.gov/gene?term=SMCC) | Several DRIP/ARC subunits are also components of other potentially related cofactors, such as CRSP, NAT, SMCC and the mouse Mediator, indicating that unique classes of activators may share common sets or subsets of cofactors. |
| 10 | [10235267](http://www.ncbi.nlm.nih.gov/pubmed/?term=10235267) | [23237](http://www.ncbi.nlm.nih.gov/sites/entrez?cmd=search&db=gene&term=23237%5Buid%5D) | [ARC](http://www.ncbi.nlm.nih.gov/gene?term=ARC) | Composite co-activator ARC mediates chromatin-directed transcriptional activation. |
| 11 | [10235267](http://www.ncbi.nlm.nih.gov/pubmed/?term=10235267) | [6046](http://www.ncbi.nlm.nih.gov/sites/entrez?cmd=search&db=gene&term=6046%5Buid%5D) | [NAT](http://www.ncbi.nlm.nih.gov/gene?term=NAT) | The ARC complex consists of 16 or more subunits; some of these are novel gene products, whereas others are present in other multisubunit cofactors, such as CRSP, NAT and mammalian Mediator. |
| 12 | [10235267](http://www.ncbi.nlm.nih.gov/pubmed/?term=10235267) | [190](http://www.ncbi.nlm.nih.gov/sites/entrez?cmd=search&db=gene&term=190%5Buid%5D) | [nuclear hormone-receptor](http://www.ncbi.nlm.nih.gov/gene?term=nuclear hormone-receptor) | Detailed analysis indicates that the ARC complex is probably identical to the nuclear hormone-receptor cofactor DRIP. |
| 13 | [10373484](http://www.ncbi.nlm.nih.gov/pubmed/?term=10373484) | [83478](http://www.ncbi.nlm.nih.gov/sites/entrez?cmd=search&db=gene&term=83478%5Buid%5D) | [p73](http://www.ncbi.nlm.nih.gov/gene?term=p73) | Here we report in vitro and in vivo analysis of homo- and hetero-oligomerization of p53 and its homologues, p63 and p73. |
| 14 | [10380929](http://www.ncbi.nlm.nih.gov/pubmed/?term=10380929) | [1000](http://www.ncbi.nlm.nih.gov/sites/entrez?cmd=search&db=gene&term=1000%5Buid%5D) | [neural cadherin](http://www.ncbi.nlm.nih.gov/gene?term=neural cadherin) | A striking organization of a large family of human neural cadherin-like cell adhesion genes. |
| 15 | [10455022](http://www.ncbi.nlm.nih.gov/pubmed/?term=10455022) | [50818](http://www.ncbi.nlm.nih.gov/sites/entrez?cmd=search&db=gene&term=50818%5Buid%5D) | [PKC](http://www.ncbi.nlm.nih.gov/gene?term=PKC) | In platelets and other secretory cells, protein kinase C (PKC) plays a role in exocytosis stimulated by physiological extracellular signals, although its linkage to the secretory machinery is poorly understood. |
| 16 | [10455022](http://www.ncbi.nlm.nih.gov/pubmed/?term=10455022) | [5870](http://www.ncbi.nlm.nih.gov/sites/entrez?cmd=search&db=gene&term=5870%5Buid%5D) | [Rab6](http://www.ncbi.nlm.nih.gov/gene?term=Rab6) | In summary, thrombin activation of platelets leads to PKC-dependent phosphorylation of Rab6 and a translocation of Rab6 to the cytosol. |
| 17 | [10455022](http://www.ncbi.nlm.nih.gov/pubmed/?term=10455022) | [1432](http://www.ncbi.nlm.nih.gov/sites/entrez?cmd=search&db=gene&term=1432%5Buid%5D) | [p38 mitogen-activated protein kinase inhibitor](http://www.ncbi.nlm.nih.gov/gene?term=p38 mitogen-activated protein kinase inhibitor) | This phosphorylation was blocked by a specific PKC inhibitor (Ro-31-8220), but not by a p38 mitogen-activated protein kinase inhibitor (PD-169316). |
| 18 | [10455115](http://www.ncbi.nlm.nih.gov/pubmed/?term=10455115) | [4982](http://www.ncbi.nlm.nih.gov/sites/entrez?cmd=search&db=gene&term=4982%5Buid%5D); [80835](http://www.ncbi.nlm.nih.gov/sites/entrez?cmd=search&db=gene&term=80835%5Buid%5D) | [TR1](http://www.ncbi.nlm.nih.gov/gene?term=TR1) | The generation of reactive oxygen species in a human carcinoma cell line was shown to result in both the oxidation of the selenocysteine in TR1 and a subsequent increase in the expression of this enzyme. |
| 19 | [10455115](http://www.ncbi.nlm.nih.gov/pubmed/?term=10455115) | [114112](http://www.ncbi.nlm.nih.gov/sites/entrez?cmd=search&db=gene&term=114112%5Buid%5D) | [TR2](http://www.ncbi.nlm.nih.gov/gene?term=TR2) | Two mammalian TR isozymes (TR2 and TR3), in addition to that previously characterized (TR1), have now been identified in humans and mice. |
| 20 | [10458166](http://www.ncbi.nlm.nih.gov/pubmed/?term=10458166) | [223](http://www.ncbi.nlm.nih.gov/sites/entrez?cmd=search&db=gene&term=223%5Buid%5D); [1738](http://www.ncbi.nlm.nih.gov/sites/entrez?cmd=search&db=gene&term=1738%5Buid%5D); [6043](http://www.ncbi.nlm.nih.gov/sites/entrez?cmd=search&db=gene&term=6043%5Buid%5D); [26765](http://www.ncbi.nlm.nih.gov/sites/entrez?cmd=search&db=gene&term=26765%5Buid%5D) | [E3](http://www.ncbi.nlm.nih.gov/gene?term=E3) | Here we describe Smurf1, a new member of the Hect family of E3 ubiquitin ligases. |
| 21 | [10491302](http://www.ncbi.nlm.nih.gov/pubmed/?term=10491302) | [7855](http://www.ncbi.nlm.nih.gov/sites/entrez?cmd=search&db=gene&term=7855%5Buid%5D); [8321](http://www.ncbi.nlm.nih.gov/sites/entrez?cmd=search&db=gene&term=8321%5Buid%5D) | [Wnt receptor](http://www.ncbi.nlm.nih.gov/gene?term=Wnt receptor) | RT-PCR analysis showed that primary endothelial and smooth muscle cell cultures, of both mouse and human origin, express members of the Wnt and Wnt receptor (Frizzled) gene families. |
| 22 | [10531035](http://www.ncbi.nlm.nih.gov/pubmed/?term=10531035) | [6016](http://www.ncbi.nlm.nih.gov/sites/entrez?cmd=search&db=gene&term=6016%5Buid%5D) | [Roc1](http://www.ncbi.nlm.nih.gov/gene?term=Roc1) | The other three subunits are the Skp1 protein; one of the cullin proteins (Cul1 in metazoans and Cdc53 or Cul A in the yeast Saccharomyces cerevisiae); and the recently identified Roc1 protein (also called Rbx1 or Hrt1). |
| 23 | [10531035](http://www.ncbi.nlm.nih.gov/pubmed/?term=10531035) | [4254](http://www.ncbi.nlm.nih.gov/sites/entrez?cmd=search&db=gene&term=4254%5Buid%5D) | [SCF](http://www.ncbi.nlm.nih.gov/gene?term=SCF) | SCF ligases bring ubiquitin conjugating enzymes (either Ubc3 or Ubc4) to substrates that are specifically recruited by the different F-box proteins. |
| 24 | [10531035](http://www.ncbi.nlm.nih.gov/pubmed/?term=10531035) | [7322](http://www.ncbi.nlm.nih.gov/sites/entrez?cmd=search&db=gene&term=7322%5Buid%5D) | [Ubc4](http://www.ncbi.nlm.nih.gov/gene?term=Ubc4) | SCF ligases bring ubiquitin conjugating enzymes (either Ubc3 or Ubc4) to substrates that are specifically recruited by the different F-box proteins. |
| 25 | [10548550](http://www.ncbi.nlm.nih.gov/pubmed/?term=10548550) | [5163](http://www.ncbi.nlm.nih.gov/sites/entrez?cmd=search&db=gene&term=5163%5Buid%5D) | [PDK1](http://www.ncbi.nlm.nih.gov/gene?term=PDK1) | SGK2 and SGK3 are activated in vitro by PDK1, albeit more slowly than SGK1, and their activation is accompanied by the phosphorylation of Thr(193) and Thr(253) respectively, the residues equivalent to the Thr in the 'activation loop' of PKB that is targeted by PDK1. |
| 26 | [10548550](http://www.ncbi.nlm.nih.gov/pubmed/?term=10548550) | [207](http://www.ncbi.nlm.nih.gov/sites/entrez?cmd=search&db=gene&term=207%5Buid%5D); [2185](http://www.ncbi.nlm.nih.gov/sites/entrez?cmd=search&db=gene&term=2185%5Buid%5D) | [PKB](http://www.ncbi.nlm.nih.gov/gene?term=PKB) | Like PKB and SGK1, SGK2 and SGK3 preferentially phosphorylate Ser and Thr residues that lie in Arg-Xaa-Arg-Xaa-Xaa-Ser/Thr motifs. |
| 27 | [10548550](http://www.ncbi.nlm.nih.gov/pubmed/?term=10548550) | [207](http://www.ncbi.nlm.nih.gov/sites/entrez?cmd=search&db=gene&term=207%5Buid%5D); [2185](http://www.ncbi.nlm.nih.gov/sites/entrez?cmd=search&db=gene&term=2185%5Buid%5D) | [protein kinase B](http://www.ncbi.nlm.nih.gov/gene?term=protein kinase B) | The catalytic domain of serum- and glucocorticoid-induced protein kinase (SGK) is 54% identical with protein kinase B (PKB) and, like PKB, is activated in vitro by 3-phosphoinositide-dependent protein kinase-1 (PDK1) and in vivo in response to signals that activate phosphatidylinositol (PI) 3-kinase. |
| 28 | [10553045](http://www.ncbi.nlm.nih.gov/pubmed/?term=10553045) | [1738](http://www.ncbi.nlm.nih.gov/sites/entrez?cmd=search&db=gene&term=1738%5Buid%5D); [3689](http://www.ncbi.nlm.nih.gov/sites/entrez?cmd=search&db=gene&term=3689%5Buid%5D) | [Lad](http://www.ncbi.nlm.nih.gov/gene?term=Lad) | Lad contains several protein-protein interaction domains including a zinc-finger motif, an SH2 domain, a proline-rich SH3 binding motif, and several phosphotyrosine sites. |
| 29 | [10607832](http://www.ncbi.nlm.nih.gov/pubmed/?term=10607832) | [1756](http://www.ncbi.nlm.nih.gov/sites/entrez?cmd=search&db=gene&term=1756%5Buid%5D) | [dystrophin](http://www.ncbi.nlm.nih.gov/gene?term=dystrophin) | To investigate this, we studied the expression of myoferlin in the mdx mouse, which lacks dystrophin and whose muscles undergo repeated rounds of degeneration and regeneration. |
| 30 | [10625670](http://www.ncbi.nlm.nih.gov/pubmed/?term=10625670) | [795](http://www.ncbi.nlm.nih.gov/sites/entrez?cmd=search&db=gene&term=795%5Buid%5D); [6277](http://www.ncbi.nlm.nih.gov/sites/entrez?cmd=search&db=gene&term=6277%5Buid%5D) | [CABP](http://www.ncbi.nlm.nih.gov/gene?term=CABP) | Five members of a novel Ca(2+)-binding protein (CABP) subfamily with similarity to calmodulin. |
| 31 | [10625670](http://www.ncbi.nlm.nih.gov/pubmed/?term=10625670) | [801](http://www.ncbi.nlm.nih.gov/sites/entrez?cmd=search&db=gene&term=801%5Buid%5D); [805](http://www.ncbi.nlm.nih.gov/sites/entrez?cmd=search&db=gene&term=805%5Buid%5D); [808](http://www.ncbi.nlm.nih.gov/sites/entrez?cmd=search&db=gene&term=808%5Buid%5D); [889](http://www.ncbi.nlm.nih.gov/sites/entrez?cmd=search&db=gene&term=889%5Buid%5D); [79823](http://www.ncbi.nlm.nih.gov/sites/entrez?cmd=search&db=gene&term=79823%5Buid%5D) | [CaM](http://www.ncbi.nlm.nih.gov/gene?term=CaM) | Five members of a novel Ca(2+)-binding protein subfamily (CaBP), with 46-58% sequence similarity to calmodulin (CaM), were identified in the vertebrate retina. |
| 32 | [10628838](http://www.ncbi.nlm.nih.gov/pubmed/?term=10628838) | [84560](http://www.ncbi.nlm.nih.gov/sites/entrez?cmd=search&db=gene&term=84560%5Buid%5D) | [MT4](http://www.ncbi.nlm.nih.gov/gene?term=MT4) | Overall, leukolysin displays the strongest homology to the newly identified MT-MMP subgroup with 45% and 39% identities to MT4- and MT1-MMPs vs 30% and 31.5% to MMP1 and 3 respectively. |
| 33 | [10628838](http://www.ncbi.nlm.nih.gov/pubmed/?term=10628838) | [3263](http://www.ncbi.nlm.nih.gov/sites/entrez?cmd=search&db=gene&term=3263%5Buid%5D) | [hemopexin](http://www.ncbi.nlm.nih.gov/gene?term=hemopexin) | Named leukolysin, it encodes for 562 residues with a conserved MMP structure, i.e. , pre-, pro-, catalytic, hinge- and hemopexin-like domains, but also a RXK/RR motif, known for its role in MMP zymogen activation, and a C-terminal hydrophobic segment. |
| 34 | [10706098](http://www.ncbi.nlm.nih.gov/pubmed/?term=10706098) | [5045](http://www.ncbi.nlm.nih.gov/sites/entrez?cmd=search&db=gene&term=5045%5Buid%5D) | [furin](http://www.ncbi.nlm.nih.gov/gene?term=furin) | The predicted protein sequence also contains a short insertion of basic residues located between the propeptide and the catalytic domain and involved in the proteolytic activation of MT-MMPs by furin-like enzymes. |
| 35 | [10706098](http://www.ncbi.nlm.nih.gov/pubmed/?term=10706098) | [3263](http://www.ncbi.nlm.nih.gov/sites/entrez?cmd=search&db=gene&term=3263%5Buid%5D) | [hemopexin](http://www.ncbi.nlm.nih.gov/gene?term=hemopexin) | The cloned cDNA encodes a protein of 562 amino acids with a domain organization similar to that of other MT-MMPs, including a prodomain with a cysteine switch, a catalytic domain with the zinc-binding site, a hemopexin-like domain, and a COOH-terminal extension rich in hydrophobic residues. |
| 36 | [10729163](http://www.ncbi.nlm.nih.gov/pubmed/?term=10729163) | [3558](http://www.ncbi.nlm.nih.gov/sites/entrez?cmd=search&db=gene&term=3558%5Buid%5D) | [lymphokine](http://www.ncbi.nlm.nih.gov/gene?term=lymphokine) | As a lymphokine, AK155 may contribute to the transformed phenotype of human T cells after infection by herpesvirus saimiri. |
| 37 | [10748143](http://www.ncbi.nlm.nih.gov/pubmed/?term=10748143) | [84892](http://www.ncbi.nlm.nih.gov/sites/entrez?cmd=search&db=gene&term=84892%5Buid%5D) | [glycosyltransferase](http://www.ncbi.nlm.nih.gov/gene?term=glycosyltransferase) | Molecular cloning of globotriaosylceramide/CD77 synthase, a glycosyltransferase that initiates the synthesis of globo series glycosphingolipids. |
| 38 | [10751307](http://www.ncbi.nlm.nih.gov/pubmed/?term=10751307) | [7508](http://www.ncbi.nlm.nih.gov/sites/entrez?cmd=search&db=gene&term=7508%5Buid%5D) | [p125](http://www.ncbi.nlm.nih.gov/gene?term=p125) | Western blotting of immunoaffinity purified calf thymus pol delta revealed the presence of p125, p50, p68 (the KIAA0039 product), and p12. |
| 39 | [10751307](http://www.ncbi.nlm.nih.gov/pubmed/?term=10751307) | [958](http://www.ncbi.nlm.nih.gov/sites/entrez?cmd=search&db=gene&term=958%5Buid%5D); [4790](http://www.ncbi.nlm.nih.gov/sites/entrez?cmd=search&db=gene&term=4790%5Buid%5D); [51008](http://www.ncbi.nlm.nih.gov/sites/entrez?cmd=search&db=gene&term=51008%5Buid%5D) | [p50](http://www.ncbi.nlm.nih.gov/gene?term=p50) | Western blotting of immunoaffinity purified calf thymus pol delta revealed the presence of p125, p50, p68 (the KIAA0039 product), and p12. |
| 40 | [10751307](http://www.ncbi.nlm.nih.gov/pubmed/?term=10751307) | [309](http://www.ncbi.nlm.nih.gov/sites/entrez?cmd=search&db=gene&term=309%5Buid%5D); [1655](http://www.ncbi.nlm.nih.gov/sites/entrez?cmd=search&db=gene&term=1655%5Buid%5D); [10657](http://www.ncbi.nlm.nih.gov/sites/entrez?cmd=search&db=gene&term=10657%5Buid%5D) | [p68](http://www.ncbi.nlm.nih.gov/gene?term=p68) | Western blotting of immunoaffinity purified calf thymus pol delta revealed the presence of p125, p50, p68 (the KIAA0039 product), and p12. |
| 41 | [10779507](http://www.ncbi.nlm.nih.gov/pubmed/?term=10779507) | [4945](http://www.ncbi.nlm.nih.gov/sites/entrez?cmd=search&db=gene&term=4945%5Buid%5D) | [OATP1](http://www.ncbi.nlm.nih.gov/gene?term=OATP1) | Cosmid clones containing the genes encoding human OATP1 (SLC21A3), OATP2 (SLC21A6), and OATP8 (SLC21A8) served to establish their genomic organization. |
| 42 | [10828014](http://www.ncbi.nlm.nih.gov/pubmed/?term=10828014) | [472](http://www.ncbi.nlm.nih.gov/sites/entrez?cmd=search&db=gene&term=472%5Buid%5D) | [TEL1](http://www.ncbi.nlm.nih.gov/gene?term=TEL1) | Additionally, the TEL2 protein is capable of associating with itself and with TEL1 in doubly transfected Hela cells, and this interaction is mediated through the pointed (PNT) domain of TEL1. |
| 43 | [10854325](http://www.ncbi.nlm.nih.gov/pubmed/?term=10854325) | [2353](http://www.ncbi.nlm.nih.gov/sites/entrez?cmd=search&db=gene&term=2353%5Buid%5D); [2354](http://www.ncbi.nlm.nih.gov/sites/entrez?cmd=search&db=gene&term=2354%5Buid%5D); [3725](http://www.ncbi.nlm.nih.gov/sites/entrez?cmd=search&db=gene&term=3725%5Buid%5D); [3726](http://www.ncbi.nlm.nih.gov/sites/entrez?cmd=search&db=gene&term=3726%5Buid%5D); [3727](http://www.ncbi.nlm.nih.gov/sites/entrez?cmd=search&db=gene&term=3727%5Buid%5D) | [AP-1](http://www.ncbi.nlm.nih.gov/gene?term=AP-1) | Interleukin-1 (IL-1) is a proinflammatory cytokine that elicits its pleiotropic effects through activation of the transcription factors NF-kappaB and AP-1. |
| 44 | [10854325](http://www.ncbi.nlm.nih.gov/pubmed/?term=10854325) | [4790](http://www.ncbi.nlm.nih.gov/sites/entrez?cmd=search&db=gene&term=4790%5Buid%5D) | [NF-kappaB](http://www.ncbi.nlm.nih.gov/gene?term=NF-kappaB) | As overexpression of Tollip results in impaired NF-kappaB activation, we conclude that Tollip is an important constituent of the IL-1R signalling pathway. |
| 45 | [10888605](http://www.ncbi.nlm.nih.gov/pubmed/?term=10888605) | [11275](http://www.ncbi.nlm.nih.gov/sites/entrez?cmd=search&db=gene&term=11275%5Buid%5D) | [Kelch](http://www.ncbi.nlm.nih.gov/gene?term=Kelch) | The sense transcript encodes a 748 amino acid protein with a predicted domain structure typical of a family of actin-organizing proteins related to the Drosophila Kelch gene, and so has been given the name Kelch-like 1 (KLHL1). |
| 46 | [10888605](http://www.ncbi.nlm.nih.gov/pubmed/?term=10888605) | [6315](http://www.ncbi.nlm.nih.gov/sites/entrez?cmd=search&db=gene&term=6315%5Buid%5D) | [SCA8](http://www.ncbi.nlm.nih.gov/gene?term=SCA8) | Spinocerebellar ataxia type 8 (SCA8) is a neurodegenerative disorder caused by the expansion of a CTG trinucleotide repeat that is transcribed as part of an untranslated RNA. |
| 47 | [10950930](http://www.ncbi.nlm.nih.gov/pubmed/?term=10950930) | [9582](http://www.ncbi.nlm.nih.gov/sites/entrez?cmd=search&db=gene&term=9582%5Buid%5D) | [cytidine deaminase](http://www.ncbi.nlm.nih.gov/gene?term=cytidine deaminase) | We have isolated the human orthologue of mouse AID cDNA, which has an open reading frame of 198 residues containing a conserved cytidine deaminase motif. |
| 48 | [10978536](http://www.ncbi.nlm.nih.gov/pubmed/?term=10978536) | [2683](http://www.ncbi.nlm.nih.gov/sites/entrez?cmd=search&db=gene&term=2683%5Buid%5D); [4635](http://www.ncbi.nlm.nih.gov/sites/entrez?cmd=search&db=gene&term=4635%5Buid%5D) | [GT1](http://www.ncbi.nlm.nih.gov/gene?term=GT1) | The polypeptide encoded by GalNAc-T9 contained the structural features characteristic of GalNAc transferases, such as a GT1 motif, a Gal/GalNAc transferase motif, (QXW)(3) repeats, and conserved His, Cys, and acidic amino acid residues. |
| 49 | [10993894](http://www.ncbi.nlm.nih.gov/pubmed/?term=10993894) | [7791](http://www.ncbi.nlm.nih.gov/sites/entrez?cmd=search&db=gene&term=7791%5Buid%5D) | [zyxin](http://www.ncbi.nlm.nih.gov/gene?term=zyxin) | Here we demonstrate that SEMA6A-1/Sema6A-1 is colocalized with EVL via its zyxin-like carboxyl-terminal domain that contains a modified binding motif, which further stresses the existence of functional differences between EVL and Mena/VASP. |
| 50 | [11056056](http://www.ncbi.nlm.nih.gov/pubmed/?term=11056056) | [2353](http://www.ncbi.nlm.nih.gov/sites/entrez?cmd=search&db=gene&term=2353%5Buid%5D); [2354](http://www.ncbi.nlm.nih.gov/sites/entrez?cmd=search&db=gene&term=2354%5Buid%5D); [3725](http://www.ncbi.nlm.nih.gov/sites/entrez?cmd=search&db=gene&term=3725%5Buid%5D); [3726](http://www.ncbi.nlm.nih.gov/sites/entrez?cmd=search&db=gene&term=3726%5Buid%5D); [3727](http://www.ncbi.nlm.nih.gov/sites/entrez?cmd=search&db=gene&term=3727%5Buid%5D) | [AP-1](http://www.ncbi.nlm.nih.gov/gene?term=AP-1) | Sequencing also revealed potential cis-acting elements for multiple transcription regulators including Sp1, GATA, C/EBP, AP-1, and Pu1. |
| 51 | [11056056](http://www.ncbi.nlm.nih.gov/pubmed/?term=11056056) | [9213](http://www.ncbi.nlm.nih.gov/sites/entrez?cmd=search&db=gene&term=9213%5Buid%5D) | [X receptor](http://www.ncbi.nlm.nih.gov/gene?term=X receptor) | No retinoic acid receptor elements or retinoic X receptor elements were detected. |
| 52 | [11058597](http://www.ncbi.nlm.nih.gov/pubmed/?term=11058597) | [4790](http://www.ncbi.nlm.nih.gov/sites/entrez?cmd=search&db=gene&term=4790%5Buid%5D) | [NF-kappaB](http://www.ncbi.nlm.nih.gov/gene?term=NF-kappaB) | IL-17E induces activation of NF-kappaB and stimulates production of the proinflammatory chemokine IL-8. |
| 53 | [11062248](http://www.ncbi.nlm.nih.gov/pubmed/?term=11062248) | [5311](http://www.ncbi.nlm.nih.gov/sites/entrez?cmd=search&db=gene&term=5311%5Buid%5D) | [PKD2](http://www.ncbi.nlm.nih.gov/gene?term=PKD2) | Finally, gastrin was found to be a physiological activator of PKD2 in human AGS-B cells stably transfected with the CCK(B)/gastrin receptor. |
| 54 | [11062248](http://www.ncbi.nlm.nih.gov/pubmed/?term=11062248) | [3005](http://www.ncbi.nlm.nih.gov/sites/entrez?cmd=search&db=gene&term=3005%5Buid%5D) | [histone H1](http://www.ncbi.nlm.nih.gov/gene?term=histone H1) | PKD2 activated by phorbol esters efficiently phosphorylated the exogenous substrate histone H1. |
| 55 | [11062248](http://www.ncbi.nlm.nih.gov/pubmed/?term=11062248) | [9874](http://www.ncbi.nlm.nih.gov/sites/entrez?cmd=search&db=gene&term=9874%5Buid%5D); [11329](http://www.ncbi.nlm.nih.gov/sites/entrez?cmd=search&db=gene&term=11329%5Buid%5D) | [serine threonine protein kinase](http://www.ncbi.nlm.nih.gov/gene?term=serine threonine protein kinase) | We have isolated the full-length cDNA of a novel human serine threonine protein kinase gene. |

1. In our error analysis on BioCreative II GN task, we manually inspected CPNM protein name tagger output and found that many protein name mentions in the text that were positively predicted by CPNM (and which appeared to be valid protein names) were actually not annotated as proteins in BioCreative II GN gold file. This led to CPNM’s higher false positive rates and thus its low precision. Some examples of such cases are shown in the table. [↑](#endnote-ref-2)
