# Supplementary material for: Context-Specific Protein Network Miner – An Online System for Exploring Context-Specific Protein Interaction Networks from the Literature
Source: PLoS One. 2012 Apr 6;7(4):e34480. doi: 10.1371/journal.pone.0034480 (PMC3321019; doi:10.1371/journal.pone.0034480)
Supplement: Table S2 — Comparison of PIMiner and NLProt. (DOC) [file pone.0034480.s003.doc]

**Table S2:** **Comparison of PIMiner and NLProt.**

| **PIMiner** | **NLProt** |
| --- | --- |
| Predicts protein interaction triplets (two protein names and one interaction word). | Does not predict protein interactions. |
| Tags protein names and interaction words in text. | Tags protein names, species/organism and tissue/cell types in text. |
| Available as web server, no installation required. | Available only as a Linux executable, and requires additional programs, such as, blastall and svm_classify. |
| PIMiner does not do protein name normalization. However, its implementation in CPNM does. | Does protein name normalization. |
| Execution time in processing 50 randomly chosen abstracts = 2 sec. | Execution time = 138 sec |
| Protein name tagging performance on AIMed dataset is: Recall: 79.0%; Precision: 68.8%; F-measure: 73.6%. | Protein name tagging performance on AIMed dataset is: Recall: 71.4%; Precision: 64.8%; F-measure: 68.0%. |
