# Supplementary material for: Context-Specific Protein Network Miner – An Online System for Exploring Context-Specific Protein Interaction Networks from the Literature
Source: PLoS One. 2012 Apr 6;7(4):e34480. doi: 10.1371/journal.pone.0034480 (PMC3321019; doi:10.1371/journal.pone.0034480)
Supplement: Table S3 — Comparison of CPNM and GNAT. (DOC) [file pone.0034480.s004.doc]

**Table S3: Comparison of CPNM and GNAT.**

| **CPNM’s protein name recognition/normalization module** | **GNAT** |
| --- | --- |
| Does protein name recognition/normalization at the sentence level. CPNM outputs normalized mentions of protein names in the context of the evidence sentence. So, basically recognition and normalization tasks are sequentially integrated in CPNM. | Has two modes for operation: i) default mode outputs a list of normalized-names that it detects in text; while ii) advanced mode can be used for tagging names in the text in the normalized forms. |
| Uses a single dictionary file with protein names from all species together. | GNAT requires running each species dictionary server separately. User needs to prepare and select his own species dictionary before running the program. |
| Accuracy of protein name normalization task on BioCreative-II GN task dataset is: Recall: 81.0%; Precision: 54.5%; F-measure: 65.2%. We had lower performance in terms of precision in part because we observed that there were some terms that appeared to be true proteins names but were not tagged as proteins by BioCreative – this reduced our precision (refer to Table S1 for more details). BioCreative-II GN evaluation task is about detecting human protein names in a set of abstracts. | Accuracy of protein name normalization task is: Recall: 71.8%; Precision: 84.1%; F-measure: 77.5%. |
| Execution time to process 262 abstracts from BioCreative-II GN task dataset = 16 sec | Execution time to process 262 abstracts from BioCreative-II GN task = 59 sec |
