# Supplementary material for: Context-Specific Protein Network Miner – An Online System for Exploring Context-Specific Protein Interaction Networks from the Literature
Source: PLoS One. 2012 Apr 6;7(4):e34480. doi: 10.1371/journal.pone.0034480 (PMC3321019; doi:10.1371/journal.pone.0034480)
Supplement: Table S4 — Comparison of CPNM and LAITOR. (DOC) [file pone.0034480.s005.doc]

**Table S4: Comparison of CPNM and LAITOR[[1]](#endnote-2)**.

|  | **CPNM** | **LAITOR** |
| --- | --- | --- |
| Design objective | Meant for extracting protein interaction networks (PINs) that can be explored using various filter functions (e.g. based on interaction word, probability). | Meant for extracting protein interactions – no explicit utility provided to draw/navigate network. |
| Usability | Process of querying PubMed abstracts and passing them to the system is *automatic*. | Process of retrieving abstracts and then passing them into the system is *manual*. |
| Usability | Has no external dependencies; various software modules are integrated into one package. | Has external dependencies; e.g. it requires external program 'NLProt'. |
| Usability | Easy to use as it is browser based; no installations required. | Relatively difficult to use; requires technical knowledge to install various Linux software. |
| Query expansion | Abstracts are retrieved from PubMed based on expanded user query (e.g. protein names in the user query are expanded by their synonyms); this option improves search coverage. | No such option available. |
| Feature | Covers for all species for which data is available; no need to generate specific dictionary that are species specific. | Currently covers only green plant and human species; requires user to generate dictionary for other species. |
| Feature | Associates predicted protein interaction (PI) triplets with evidence sentence. | No such feature. |
| Feature | Predicts directionality of protein interactions. | No such feature. |
| Feature | Generates protein interaction networks in real time. | No such feature. |
| Feature | Summarizes basic network topology properties. | No such feature. |
| Overall Workflow | Input to CPNM (user search keywords) -> Output (PINs). Protein interaction prediction is just an intermediate step. | Input to LAITOR (entity tagged sentences) -> Output (list of all candidate/putative protein interaction triplets that are present in a sentence); it does not attempt to predict the true ones. |

1. Overall, LAITOR is a system that has some level of similarity to CPNM. However, it offers much reduced set of features and provides reduced information output. Moreover, the design objectives of the two systems are very different (refer Figure S1 for a sample output from LAITOR that we obtained after executing the program on a small set of randomly chosen abstracts). [↑](#endnote-ref-2)
